# Ocular manifestations and full house membranous nephropathy as a rare presentation of secondary syphilis

**DOI:** 10.1016/j.idcr.2022.e01461

**Published:** 2022-03-02

**Authors:** Ellen Ann Sockman, Jordan Guffey, Joel Yednock, Melanie Fisher

**Affiliations:** West Virginia University, Morgantown, WV, USA

**Keywords:** ANA, antinuclear antibody, RPR, rapid plasma reagin, VDRL, venereal disease research laboratory test, IgA, immunoglobulin A, IgG, immunoglobulin G, IgM, immunoglobulin M, C1q, complement component 1q, HIV, human immunodeficiency virus, CSF, cerebrospinal fluid, ANCA, antineutrophil cytoplasmic antibodies, Syphilis, Secondary syphilis, Membranous nephropathy, Full house membranous nephropathy and syphilis, Rare manifestations of syphilis

## Abstract

Syphilis is an often-overlooked diagnosis and without timely diagnosis and treatment, can have serious repercussions. Although its prevalence had decreased with the introduction of penicillin, it has had a resurgence over the years. Discerning the proper patient population to test for syphilis should be led by a patient’s risk factors. Here, we present a patient diagnosed with secondary syphilis, with initial concern for a possible concomitant lupus diagnosis. He initially presented with visual symptoms and optic nerve inflammation, along with a positive antinuclear antibody (ANA). Due to an unprotected sexual encounter, there was suspicion for a sexually transmitted infection. Testing revealed reactive rapid plasma reagin (RPR) (≥1:256 titer) and reactive treponemal antibody, consistent with active syphilis. He was immediately started on intravenous Penicillin G. Lumbar puncture was consistent with a reactive venereal disease research laboratory test (VDRL). Urinalysis revealed nephrotic range proteinuria, which along with the positive ANA, prompted renal biopsy. This showed membranous nephropathy with full house staining, which is seen primarily in lupus nephritis and further confounded the diagnosis. He completed a two-week course of penicillin and steroids inpatient with clinical improvement. On follow up, his RPR improved (≥1:64 titer), and lumbar puncture showed a non-reactive VDRL. Due to the resolution of proteinuria, decrease of the ANA titer and no further positive testing or symptoms convincing for a concomitant rheumatologic disorder, the presence of lupus was collectively determined to be of low concern. and the sole diagnosis of secondary syphilis was made.

## Highlights

• Ocular symptoms and membranous nephropathy as a presentation of secondary syphilis.
• Secondary syphilis presenting with a full house staining pattern on renal biopsy.
• Diagnostic challenge with positive autoimmune labs confounding the diagnosis.

## Introduction

### Case report

Syphilis is known as one of the great mimickers, as it may manifest with symptoms that overlap with many diseases, especially autoimmune disorders such as lupus. It is also an often-overlooked diagnosis, secondary to its previously low prevalence, although it has been making a resurgence in recent years. Although syphilis has been known to present with secondary membranous nephropathy, a full house staining pattern (presence of IgA, IgG, IgM and C1Q deposits on immunofluorescence) on renal biopsy is typically exclusive to lupus nephritis [1]. Here we present a challenging case in which our patient who presented with ocular symptoms and a full house staining pattern was ultimately diagnosed with secondary syphilis, although there was a lingering question if a concomitant diagnosis of lupus was present.

### Case description

A 54-year-old male with no significant history presented to his primary care provider with a multitude of symptoms that occurred over several months which included tinnitus, a torso rash, intermittent joint pain and swelling, bloodshot puffy eyes, foamy urine, and left groin lymphadenopathy. Initial work up was significant for a positive speckled antinuclear antibody (ANA) [1:640] and an elevated c-reactive protein [33.4 mg/L]. His complete blood count, basic metabolic panel, and hepatic function panel were otherwise within normal limits, including creatinine. Other pertinent negative labs included: Lyme antibody, anti-cyclic citrullinated peptides (anti-CCP), hepatitis panel, chlamydia, gonorrhea, and human immunodeficiency virus (HIV) antigen/antibody. Due to concerns for an underlying autoimmune process, he was referred to outpatient dermatology and rheumatology.

Two weeks later, he noticed a dark spot and black lines in his vision and presented to the ophthalmology clinic. Retinal exam noted inflammation of the optic nerve and petechial hemorrhages in the conjunctiva. They recommended admission due to concern for lupus papillitis, he was admitted to our service on IV methylprednisolone 250 mg every six hours and evaluated with further history and work up. He had no history of prior immunodeficiency, recent travel, sick contacts, or intravenous drug use. He admitted to a single unprotected sexual encounter of intercourse with a female partner four months prior. He did not admit to any further risky sexual behaviors including multiple partners, oral sex, or sex with male partners. Due to this recent encounter, our suspicion was increased for a sexually transmitted infection and further testing was pursued.

Physical examination showed a well-appearing male with no cardiac or lung abnormalities. Abdominal examination was benign. No visible rashes were present at the time of admission. Left inguinal lymphadenopathy was present. No abnormalities were noted on genitourinary examination with no presence of chancres. Vital signs were unremarkable.

Complete blood count showed minor leukocytosis and reactive thrombocytosis [hemoglobin 13.6 g/dL, white blood cells 11.5 × 103 /µL, platelets 436 × 103/µL). Basic metabolic panel was without abnormality [sodium 130 mmol/L, potassium 4.0 mmol/L, creatinine 0.64 mL/dL]. The hepatic function panel was normal except for low albumin [aspartate aminotransferase 20 U/L, alanine transaminase 19 U/L, total protein 7.6 g/dL, albumin 2.5 g/dL]. HIV and hepatitis C screening tests were negative. ANA was positive and speckled [1:320], C4 [10 mg/dL], C3 [91 mg/dL], c-reactive protein [38.9 mg/L], sedimentation rate [74 mm/hr]. Urinalysis was consistent with nephrotic range proteinuria - cloudy urine with> 500 mg/dL protein, random urine protein [1277 mg/dL, urine creatinine 81 mg/dL], with a urine protein to creatinine ratio of 15.7 mg/dL. Due to ocular involvement, imaging was performed. Magnetic resonance imaging of the brain was consistent with a single punctate focus of T2/flair intensity in the parasagittal left occipital lobe, which was noted to be nonspecific, with no intracranial enhancing lesions present.

He was continued on the IV methylprednisolone as results were pending and specialists provided recommendations. On the second day of hospitalization, the reactive RPR [≥ 1:256] and reactive treponemal antibody resulted, consistent with active syphilis. He was immediately started on IV Penicillin G 4 million units every four hours with plans to do a lumbar puncture. Due to the presence of an infectious process and not wishing to suppress its treatment, the steroids were transitioned to oral prednisone 60 mg daily with plans to taper. However, due to concerns for concomitant lupus nephritis secondary to the initial positive ANA and nephrotic range proteinuria, a renal biopsy was recommended by nephrology and was performed on day three.

**Fig. 1:**
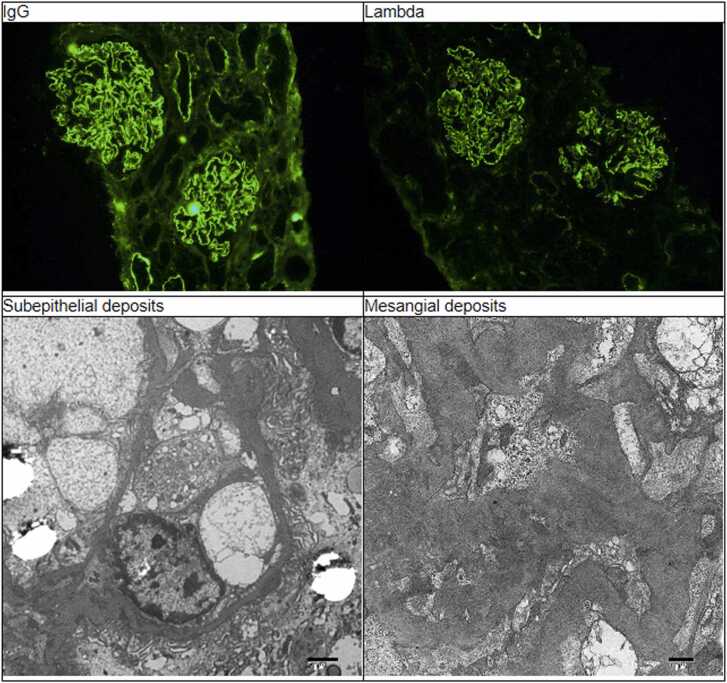
Renal biopsy immunofluorescence and electron microscopy showing membranous nephropathy, with C1q staining positive and full house staining. Staining was negative for phospholipase A2 Receptor, thrombospondin, IgG4 and EXT1. Warthin starry stain did not show any treponemal organisms in the tissue.

Renal biopsy was consistent with membranous nephropathy, with C1q staining positive and near full house staining (Fig. 1). Warthin starry stain did not show any definite treponemal organisms in the tissue, and additional stains sent to an outside lab for treponemal antibodies were negative. Electron microscopy noted severe foot process effacement of the podocytes, with many subepithelial immune type electron dense deposits, along with mesangial and para-mesangial deposits. All the above findings supported a diagnosis of secondary membranous nephropathy, with a suggested etiology of syphilis vs class V lupus nephritis. However, due to the patient’s age and diagnosis of active syphilis, we were not convinced there was an underlying previously undiagnosed autoimmune disorder and were diligent to assess this further.

Due to initial difficulty with an attempted LP on hospital day four due to complex anatomy from prior surgery, followed by limitations scheduling with interventional radiology, the patient underwent a lumbar puncture on hospital day five. The cerebrospinal showed a reactive VDRL [1:8], protein [84], glucose [70], culture with no growth and no organisms. The sample was xanthochromic and cloudy, likely secondary to peripheral blood contamination as well as lymphocytic pleocytosis secondary to infection or inflammation [37 nucleated cells, 4530 red blood cells, 20% neutrophils, 73% lymphocytes, 7% monocytes].

Rheumatology was consulted during the admission due to the concern for underlying concomitant lupus. Further autoimmune and infectious testing was ultimately negative other than those noted above, and included anti-smith, anti-double stranded DNA (dsDNA), antineutrophil cytoplasmic antibody panel, proteinase, beta-2 glycoprotein, cardiolipin antibody, C3, C4, parvovirus B19 antibody IgM, immunoglobulin G4 antibody, serum protein electrophoresis, neuromyelitis optica antibody, and angiotensin-converting enzyme. Their suspicion remained ambiguous if there was a secondary diagnosis of lupus present and agreed with tapering the patient off steroids while completing treatment for syphilis, with outpatient follow up and repeat testing.

He remained inpatient for approximately a two-week period, in which he received a total of 14 days of IV Penicillin G 4 million units every four hours. The prednisone was tapered throughout admission, and he was discharged on 10 mg/day to continue for a total of 21 days of steroid therapy.

One week after discharge, repeat urinalysis showed complete resolution of proteinuria, and remained clear on follow up at three and four months. His renal function remained within a normal range through the entirety of his disease course. Due to the resolution of the proteinuria, a repeat renal biopsy was not pursued.

Repeat RPR testing was performed two months after discharge and remained reactive [≥ 1:256], but at four months showed improvement [1:64]. Rheumatology repeated autoimmune labs three months later which were consistent with a now homogenous, lower ANA [1:160], normal C3, C4 and negative anti-smith and anti-dsDNA. His joint pain, rash, and visual symptoms resolved. Follow up retinal examination showed resolution of his optic nerve inflammation. Six months after discharge, a repeat lumbar puncture showed a non-reactive VDRL, protein [45], glucose [62], culture with no growth, no polymononuclear leukocytes or organisms identified. The sample was colorless and clear [3 nucleated cells, 1 red blood cell]. Repeat HIV testing remained negative. All testing was completed by the same laboratory.

Due to his clinical improvement, resolution of the proteinuria, and no further symptomatic or laboratory manifestations of an autoimmune condition, the multiple subspeciality teams concluded secondary syphilis was the cause of the patient’s presentation and had a very low suspicion for a concomitant lupus diagnosis.

## Discussion

Syphilis is a sexually transmitted infection caused by the spirochete, *Treponema pallidum*. Over the years, the prevalence of syphilis had historically been decreasing with the introduction of penicillin. Unfortunately, however, the rate of infection has been steadily increasing over the past twenty years with approximately 55,400 people newly infected with syphilis in the US each year [2]. Syphilis is most commonly present in patients co-infected with human immunodeficiency virus and should be high on the differential when patients present with risk factors including engaging in unprotected sexual intercourse, oral sex, having multiple partners, or in populations such as men who have sex with men [3]. In the case of our patient, our suspicion for a sexually transmitted infection was increased secondary to his unprotected sexual encounter several months prior. Our case highlights the importance of maintaining a high index of suspicion when any of the forementioned risk factors are present, as a delay in the diagnosis and treatment of syphilis can result in organ threatening and potentially long-term complications.

One of the main confounding features of our case which concerned us for a dual diagnosis of syphilis and lupus were the results of the renal biopsy. Although membranous nephropathy has been previously connected with the presentation of syphilis [2], the full house staining pattern elicited on our patient’s biopsy has typically only been seen in lupus nephritis, and is described as the presence of IgA, IgG, IgM and C1Q deposits on immunofluorescence [1]. After performing a literature review, we were able to locate only a single case report in which a patient presented with syphilis and parvovirus B19 co-infection imitating lupus nephropathy, demonstrating the full house staining pattern [1]. Typically, when syphilis presents as a membranous nephropathy, it is easy to discern from lupus membranous nephropathy based on the presence of immune deposits, namely the deposition of C1Q which is pathognomonic of lupus nephritis [4]. There have also been reports of “pseudo-lupus nephritis” with other conditions, such as HIV infection or infectious endocarditis [5], [6], [7]. Therefore, our patient’s renal biopsy presentation of syphilis appears to be quite rare.

Throughout the course of our patient’s case, our suspicion for lupus nephritis decreased due to the lack of antibody testing and lack of clinical signs/symptoms. It is well known that anti-dsDNA antibodies appear to be of primary importance to the pathogenesis of lupus nephritis. There is a known association which exists between anti-dsDNA antibodies and active glomerulonephritis, as well as evidence of dsDNA containing immune complex deposition within the glomeruli [8]. In the case of our patient, he tested negative for anti-dsDNA antibodies on his initial presentation, as well as on follow up several months later.

## Conclusion

Ultimately this case illustrates the importance of eliciting a proper history, along with a targeted work up based on the patient’s risk factors and presentation. Syphilis diagnosis is often delayed, particularly that of ocular syphilis, and suspicion should remain high when patients exhibit certain risk factors. As evidenced by this case, the proper and timely diagnosis and treatment led to a rapid improvement in symptoms and lab abnormalities associated with the disease and prevented further disease progression and complications.

## Funding

None.

## Ethical approval

Not required, no studies were performed on patients.

## Author contribution

**Ellen Ann Sockman:** Writing. **Jordan Guffey:** Writing. **Joel Yednock:** Editing. **Melanie Fisher:** Editing.

## Consent

Written informed consent was obtained from the patient for publication of this case report and accompany in gimages. A copy of the written consent is available for review by the Editor-in-Chief of this journal on request.

## Declaration of Competing Interest

The authors declare that they have no known competing financial interests or personal relationships that could have appeared to influence the work reported in this paper.
